# A Case of Unusual Speech Defect

**Published:** 1896-06

**Authors:** 


### A Case of Unusual Speech Defect.

Dr. G. Hudson Makuen, of Philadelphia, presented a young law student who had a marked retraction of the lower jaw, which destroyed the character of the labial sounds. This could only be overcome, he said, by long practice in protruding the lower jaw when speaking. The soft palate was much relaxed, and had been impeded in its action by the existence of adenoid growths. In trying to say "s," instead of the palate rising to the roof of the mouth, it remained down on the tongue. He considered the adenoid thickening in the pharynx as the original cause of the defect in the speech. The patient had received careful instruction as to the best method of overcoming the difficulty of speech, and he demonstrated clearly the marked improvement that had already resulted from persistent practice along this line.

